# Doxorubicin-Loaded PEG-CdTe Quantum Dots as a Smart Drug Delivery System for Extramedullary Multiple Myeloma Treatment

**DOI:** 10.1186/s11671-018-2782-0

**Published:** 2018-11-22

**Authors:** Dangui Chen, Bing Chen, Fusheng Yao

**Affiliations:** 1 Department of hematology, Anqing Municipal Hospital, Anqing Hospital Affiliated to Anhui Medical University, Anqing, 246003 People’s Republic of China; 2 Department of hematology, Nanjing Drum Tower Hospital, The Affiliated Hospital of Nanjing University Medical School, Nanjing, 210008 People’s Republic of China

**Keywords:** PEG-CdTe QDs, Nanoparticles, Doxorubicin, Extramedullary multiple myeloma

## Abstract

New drug treatments still do not improve the prognosis of extramedullary multiple myeloma (EMM) patients. Luckily, high-dose chemotherapy can raise the prognosis, but is intolerant to most patients because of drug cytotoxicity. Nanoparticles (NPs) are used as drug carriers to prolong drug circulation time, control drug release, reduce drug toxicity and bioavailability, and target specific sites. In this work, doxorubicin (DOX) was loaded in polyethylene glycol-modified cadmium telluride quantum dots (PEG-CdTe QDs). PEG-CdTe-DOX facilitated intracellular drug accumulation through polyethylene organizational compatibility and released DOX into the microenvironment in a pH-controlled manner, which enhanced the therapeutic efficacy and the apoptosis rate of myeloma cells (PRMI8226). PEG-CdTe-DOX improved the anti-tumor activity of DOX by regulating the protein expressions of apoptosis-associated genes. In summary, PEG-CdTe-DOX provides a specific and effective clinical treatment for EMM patients.

## Introduction

Multiple myeloma (MM), the second largest hematologic tumor after lymphoma [1], is a malignant neoplasm of plasma cells accumulated in the bone marrow and leads to bone destruction and marrow failure. Life expectancy of myeloma patients has been prolonged significantly in the last 10–20 years thanks to the development of more effective chemotherapeutic agents and regimens with high anti-tumor activity [2, 3]. Extramedullary multiple myeloma (EMM), defined as the presence of plasma cells outside the bone marrow of multiple myeloma patients, may account for up to 30% of multiple myeloma across the overall disease course [4]. In addition to the poor prognosis, the median overall survival of EMM patients suffering extramedullary relapse is < 6 months [4]. Concerning the use of novel agents, none of 11 EMM patients reportedly responded to single-agent thalidomide, while 16 of 27 patients without extramedullary involvement responded [5]. Moreover, even new-drug-based treatment, such as bortezomib, does not improve the survival of EMM patients [6]. Yet, some studies show that high-dose chemotherapy can promote the prognosis of EMM patients [7, 8]. However, the high dosage of chemotherapeutic poorly tolerated by patients results in significant side effects in normal tissues and organs.

Doxorubicin (DOX) is one of the most effective chemotherapeutic drugs for treatment of multiple myeloma [6, 9]. However, its high-dose clinical applications into the elderly or EMM patients are seriously limited by its toxicity and side effects, mainly including cardiotoxicity and bone marrow suppression. In addition, myeloma is most frequently diagnosed among people aged 65 to 74 years, with the median of 69 years [9]. Indeed, chemotherapy is still the main treatment of multiple myeloma, but chemotherapeutic drugs often cause irreversible damage to normal tissues or organs while killing tumor cells and reducing the body immune capacity. Thus, traditional chemotherapy cannot achieve the desired effect. Minimizing the adverse effects of chemotherapy is still challenging. Statistics show the annual average rate of new myeloma has increased by 0.8% over the past decade [9]. Meanwhile, there is no standard treatment plan for EMM patients. Therefore, new treatments for EMM patients are urgently needed.

To overcome the limitations of conventional chemotherapeutic treatments, researchers have mainly used liposomal carfilzomib nanoparticles could effectively target multiple myeloma cells [10]. DOX-loaded platelets can enhance the therapeutic effectiveness for lymphoma [11]. Nanoparticles (e.g., cadmium telluride quantum dots or CdTe QDs) can prolong circulation time and drug release in a pH-controlled manner, improve efficacy, and reduce drug toxicity [12–16]. Polyethylene glycol (PEG), featured by hydrophilicity, high biocompatibility, safety, non-toxicity, and low immunogenicity, can be conjugated with CdTe QDs (PEG-CdTe QDs) to induce an “immune-neglected” effect, reducing or even avoiding plasma opsonization and absorption by the reticuloendothelial system [14]. This property contributes to prolonging the blood circulation time of PEG by minimizing or eliminating the adsorption of plasma proteins on these particles [17]. PEG-CdTe QDs are efficiently internalized by cells through the fluid phase endocytosis and the lipid bilayer affinity on the plasma membrane surface [18]. As a drug nanoparticles vehicle, drugs loaded CdTe QDs can release drugs in a pH-controlled and pH-triggered pattern, and these nanoparticle complexes can release drugs at low pH that is similar to the tumor microenvironment [13].

**Fig. 1:**
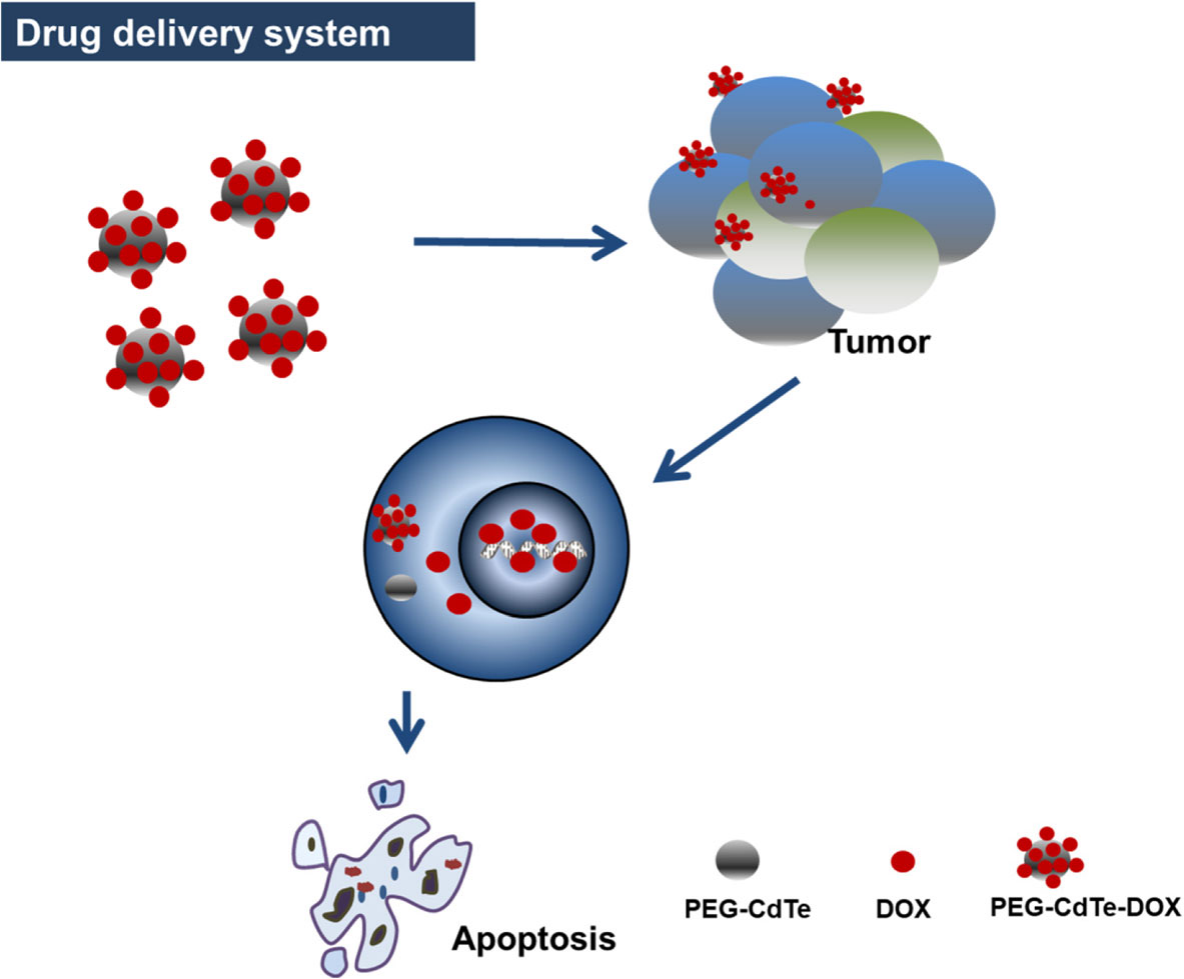
Schematic illustration of possible mechanism of improved anti-tumor activity of PEG-CdTe-DOX in vitro. Notes: they can be internalized when targeting tumor cells through high tissue compatibility of PEG. DOX induces tumor cell apoptosis through regulating the expression of apoptosis-associated genes

In this study, a nanoparticle drug delivery system (PEG-CdTe-DOX) was synthesized to improve the chemotherapeutic efficacy. This system facilitated the preferential delivery of DOX into PRMI 8226 cells. The schematic map of the system synthesis and function is shown in Fig. 1. The drug delivery system was characterized, and its anti-tumor effects and systematic toxicity were tested in vitro. This study may provide new ideas for EMM treatment.

## Materials and Methods

### Materials

PEG-CdTe QDs and Pierce™ bicinchoninic acid (BCA) protein assay kit were purchased from Sigma-Aldrich (St. Louis, USA). DOX was gained from Dalian Meilun Biology Technology Co. (Dalian, People’s Republic of China). PRMI-1640 was gained from Gibco Chemical Co. (Carlsbad, CA, USA). Fetal bovine serum (FBS) was purchased from Wisent Inc. (Montreal, Canada). Annexin V-Fluorescein isothiocyanate (FITC) apoptosis detection kit and cell counting kit-8 (CCK-8) assay were obtained from Beyotime Biotechnology Co., Ltd. (Nantong, People’s Republic of China), and monoclonal antibodies to Bax, Caspase-3, Bcl2, β-actin were gained from Cell Signaling Technology, plc. (Boston, USA). A transmission electron microscope (TEM) was accepted by a Japan Electron Optics Laboratory, Ltd. (Tokyo, HT700, Japan). The hydrodynamic diameters and sized distribution of PEG-CdTe and PEG-CdTe-DOX were measured by Zetasizer sizer NanoZs size analyzer (Malvern, Zetasizer Ultra, UK). The concentration of DOX was detected by HPLC (Jasco, LC-2000, Japan). The DOX delivery has found by Laser scanning confocal microscope (Nikon, C 2, Japan). The apoptosis and fluorescence intensity of PRMI 8226 cells by flow cytometer (BD, Biosciences Accuri C6, USA). Protein blots were detected using the ECL detection system (Tanon, 5200 Multi, China).

### Preparation of PEG-CdTe-DOX

DOX was absorbed onto PEG-CdTe QDs via electrostatic interaction efficiently. Briefly, 100 μl of PEG-CdTe (1 mg/ml) and 100 μl of DOX (1 mg/ml) were mixed in 800 μl of distilled water under stirring of 100 rpm for 24 h at 37 °C in the dark. PEG-CdTe-DOX was collected and centrifuged for 30 min (4 °C, 30,000 rpm). PEG-CdTe-DOX was mixed and centrifuged twice at the above mentioned. The unreacted DOX were tested by high-performance liquid chromatography (HPLC) [19]. Encapsulation efficiency (EE) and drug loading (DL) were calculated as follows:

$$ \mathrm{DL}=\frac{\mathrm{Weight}\ \mathrm{of}\ \mathrm{the}\ \mathrm{drug}\ \mathrm{in}\ \mathrm{NPs}}{\mathrm{Weight}\ \mathrm{of}\ \mathrm{the}\ \mathrm{NPs}}\times 100\% $$

$$ \mathrm{EE}=\frac{\mathrm{Weight}\ \mathrm{of}\ \mathrm{the}\ \mathrm{drug}\ \mathrm{in}\ \mathrm{NPs}}{\mathrm{Initial}\ \mathrm{weight}\ \mathrm{of}\ \mathrm{the}\ \mathrm{drug}}\times 100\% $$

The morphology of PEG-CdTe QDs was characterized by TEM. The hydrodynamic diameters of PEG-CdTe and PEG-CdTe-DOX were measured by dynamic light scatting (DLS) in the PBS. In brief, 20 ml of PEG-CdTe-DOX (DOX, 1 mg/ml) was transferred in dialysis bags (molecular weight cutoff of 3500 Da), which were then immersed in 180 ml of PBS at pH 5.0, 6.0,7.4 or 8.0, under constant turning (at 100 rpm) at 37 °C. Then, 0.1 ml of PBS was collected every 2 h and replaced with an equivalent volume of PBS. The DOX concentration was quantified by spectrophotometry at 450 nm, and the cumulative release of DOX from the PEG-CdTe was plotted according to the release ratio over time.

### Confocal Fluorescence Microscopy

The entrance of DOX into PRMI 8226 cells (multiple myeloma line) was confirmed visually under confocal microscopy. The PRMI 8226 cells were treated with PEG-CdTe, DOX, or PEG-CdTe-DOX for 4 h. Then, the PRMI 8226 cells were collected, centrifuged, and suspended in 70 μl of PBS. Then, the cells were transferred to glass slides. After 4′,6-diamidino-2-phenylindole (DAPI) for 10 min, confocal fluorescence images were taken with a confocal inverted microscope. The emission wavelength of DAPI and DOX were 450 and 585 nm, respectively.

### Cell Culture

PRMI 8226 (multiple myeloma cells) and 293 t (human embryonic kidney cells) cell lines were purchased from Shanghai Institute of Cells (Chinese Academy of Sciences, Shanghai, China). PRMI 8226 cells were cultured in a Roswell Park Memorial Institute (RPMI)-1640 medium supplemented with 10% FBS, 293 t cells were cultured in a Dulbecco’s modified Eagle’s medium (DMEM) medium supplement with 10% FBS, and 100 U/ml penicillin, and 100 μg/ml streptomycin at 37 °C in a humidified atmosphere with 5% carbon dioxide (CO_2_).

### Cell Viability Assay

Cell viability was evaluated by the CCK-8 assay. Firstly, PRMI 8226 cells were seeded in 96-well plates each containing 100 μl of 2 × 10^4^ cells and treated with phosphate buffer saline (PBS) (the control group), PEG-CdTe, DOX, or PEG-CdTe-DOX. The DOX concentration was 1.76 μg/ml after incubation for 24, 48, or 72 h at 37 °C in a humidified atmosphere with 5% CO2. 293 t cells were seeded in 96-well plates each containing 100 μl of 8 × 10^4^ cells and treated with PEG-CdTe (0, 0.4, 0.8, 1.6, 3.2, 6.4, 12.8, 25.6 or 51.2 μg/ml) for 24 h. Then, CCK-8 (10 μl) was added into 96-well plates and incubated for another 3 h. Cell viabilities (%) of PRMI 8226 cells and 293 t were calculated as (1 − OD_treatment_/OD_control_) × 100.

### Cellular Uptake

Cellular uptake was quantitatively measured by flow cytometry (FCM) to determine if the intracellular DOX concentration increases in PEG-CdTe-DOX treated cells (Fig. 3). Briefly, PRMI 8226 cells were cultured with PEG-CdTe, DOX, or PEG-CdTe-DOX in 24-well plates and incubated for 24 h. The cells were centrifuged and washed two times at 1000 rmp for 5 min. The precipitate was dispersed in 200 μl of PBS and analyzed by FCM. The anto-fluorescence of DOX is red, and the relative fluorescence intensity (FI) was calculated as FI_treated cells_/FI _PEG-CdTe cells_.

### Cell Apoptosis Study Test

PRMI 8226 cells were seeded in 24-well plates at a density of 3 × 10^5^ cells. After incubation with PBS, PEG-CdTe, DOX, or PEG-CdTe-DOX for 24 h, these cells were gathered through centrifugation at 1000 rpm, and were washed three times with PBS. Cells in different groups were labeled with Annexin V-FITC (5 μl) in binding buffer (100 μl) for 15 min, followed by addition of 5 μl of PI for 5 min and in the dark room. Cell apoptosis rate was measured by FCM.

### Western Blotting

PRMI8226 cells were harvested after different treatments (PBS, PEG-CdTe, DOX, or PEG-CdTe-DOX) and subjected to western blotting. Total proteins were collected from all groups and the protein concentration was detected by the Braford method. Total proteins (40 μg) were size-fractionated by 10% sodium dodecyl sulfate polyacrylamide gel electrophoresis (SDS/PAGE) and transferred to polyvinylidene difluoride membranes. Then, 5% non-fat milk was used as a blocking agent for 1 h at room temperature. Western blotting was performed using monoclonal antibodies: β-actin (internal control), Bax, Bcl2, Caspase-3. Protein blots were detected using the ECL detection system, and protein band was quantified by Image J.

### Statistical Analysis

All values were given as means ± standard deviation. Differences between two groups analyzed by Student’ *t* test. Values of *P* < 0.05 were considered statistically significant. All experiments were performed three repetitions at least.

## Results

### Characterization of PEG-CdTe-DOX

**Fig. 2:**
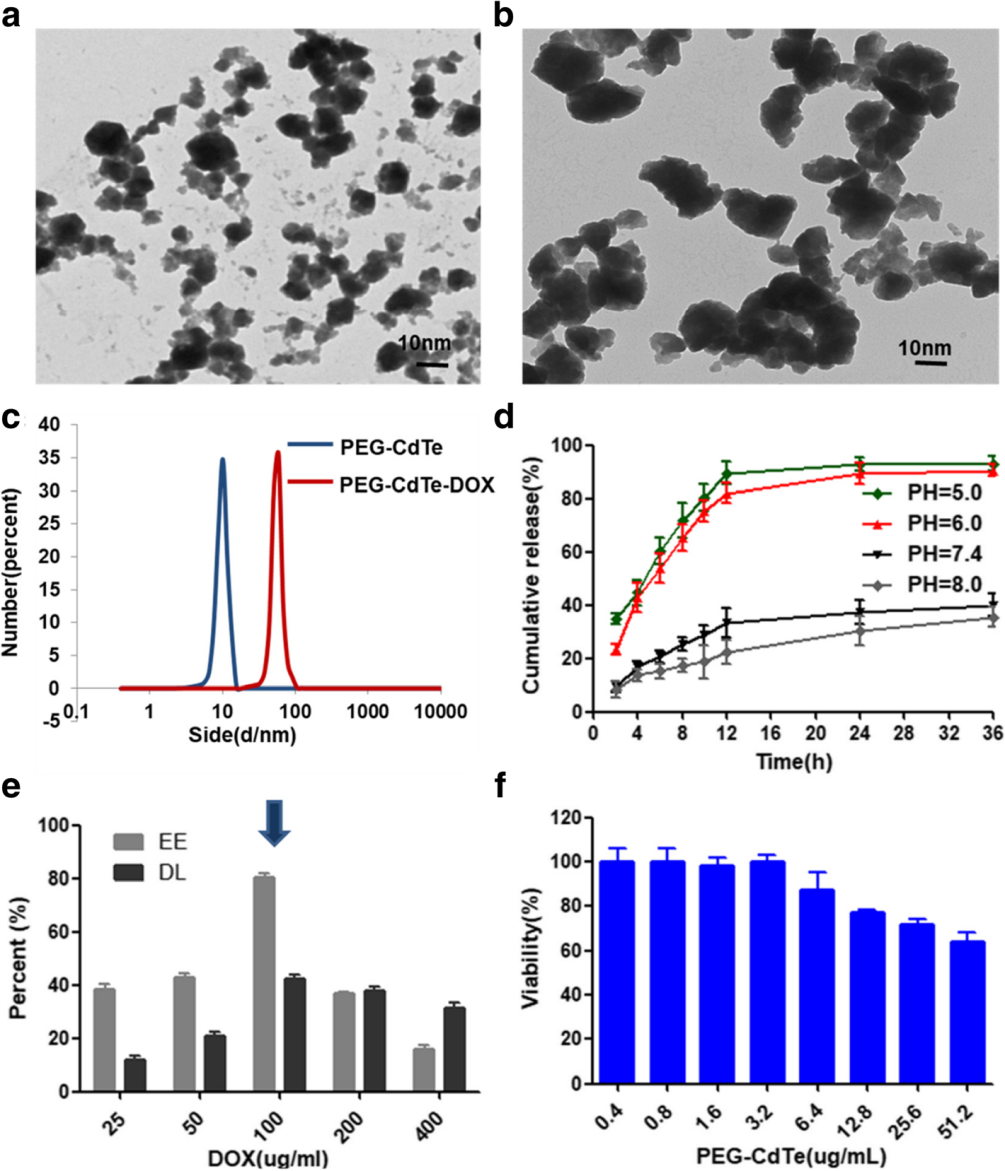
Characteristics of PEG-CdTe and PEG-CdTe-DOX. Notes: **a**, **b** TEM images of PEG-CdTe and PEG-CdTe-DOX. **c** DLS analysis of PEG-CdTe and PEG-CdTe-DOX. **e** The optimized PEG-CdTe-DOX drug delivery system synthesizing from different incubation concentrations of DOX to PEG-CdTe. **d** DOX released from PEG-CdTe-DOX in PBS at pH 5.0, 6.0, 7.4, or 8.0 at 37 °C in vitro. **f** The viability of 293 t cells after treatment with different concentrations of PEG-CdTe

PEG-CdTe and PEG-CdTe-DOX exhibited a crystal structure and dispersed well in PBS, as detected by TEM (Fig. 2a, b). The nanoparticle size distributions of PEG-CdTe and PEG-CdTe-DOX were determined by dynamic light scattering (DLS) (Fig. 2c). The DL and EE of DOX in PEG-CdTe-DOX were determined by HPLC (Fig. 2e). The DOX release maximized at pH 5.0 and 6.0, as about 92.5% and 88% of the loaded DOX was released into the PBS within 24 h, and the release rates were slower at pH 7.4 and 8.0, which suggested a pH-triggered release pattern (Fig. 2d). The pH-sensitive releasing ability of DOX is effective because an acidic environment is formed in the tumor microenvironment, in which DOX is rapidly released from the PEG-CdTe-DOX complex. The average hydrodynamic diameters are 8.20 and 78.31 nm, respectively; the EE and DL are 77.20% ± 1.12% and 42.12% ± 0.98%, respectively; the Zeta potentials of PEG-CdTe and PEG-CdTe-DOX are − 20.12 ± 2.45 and − 10.50 ± 1.26 mV, respectively. These findings indicate the favorable loading of DOX on PEG-CdTe and the successful synthesis of PEG-CdTe-DOX. Hence, higher DOX concentration in the tumor microenvironment and lower DOX concentration in normal tissues could be achieved.

Confocal microscope fluorescence images show PEG-CdTe-DOX could deliver DOX into PRMI 8226 cells. Moreover, red fluorescence markedly stored in PRMI 8226 cells the PEG-CdTe-DOX group compared to the DOX group indicating PEG-CdTe could strengthen the cellular uptake of PEG-CdTe-DOX and intracellular delivery by targeting RPMI 8226 cells.

### Cellular Uptake and Cytotoxicity of PEG-CdTe-DOX In Vitro

**Fig. 3:**
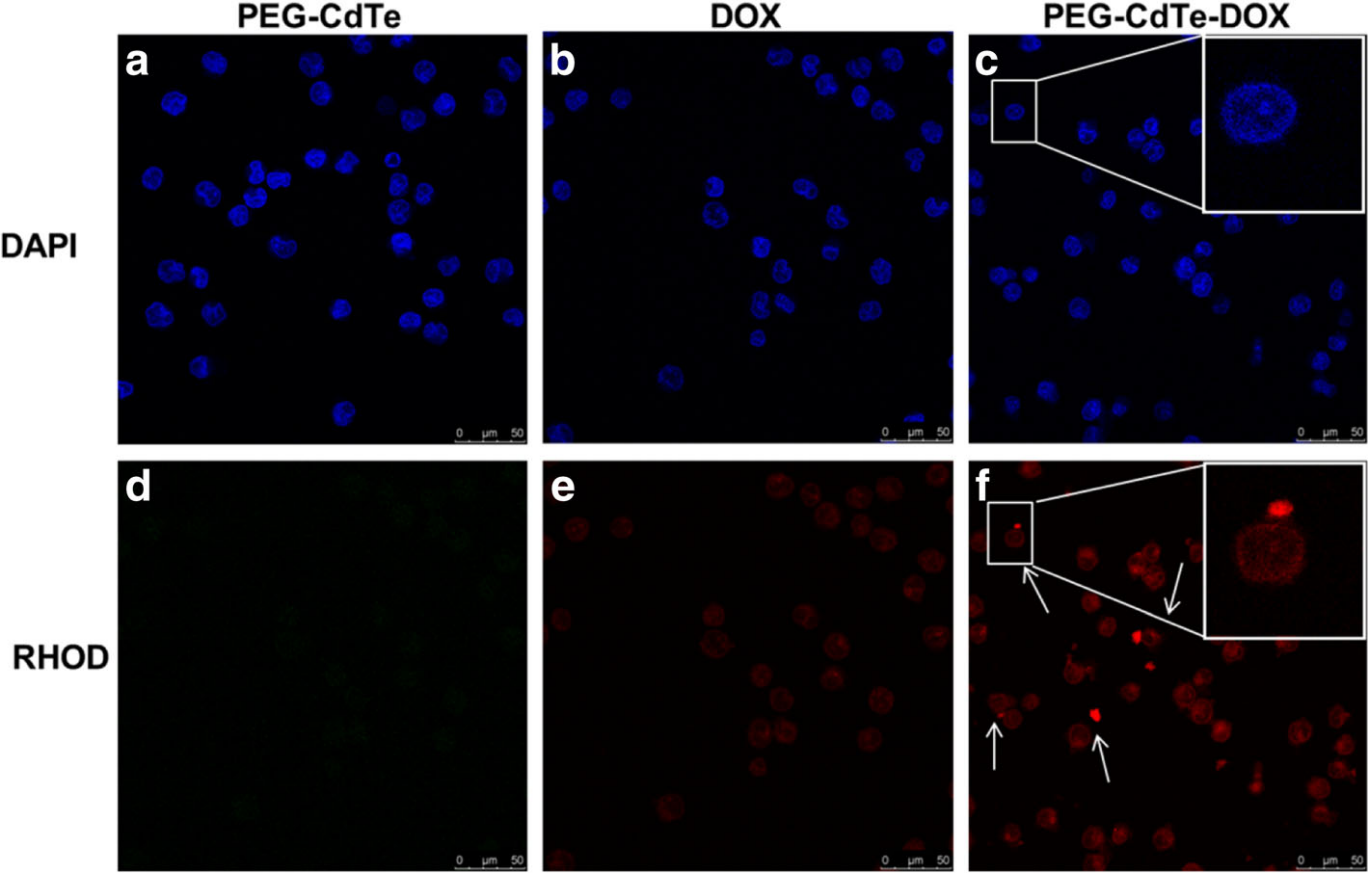
Fluorescence microscopy images of PRMI 8226 cells after receiving PEG-CdTe, DOX, or PEG-CdTe-DOX. Notes: **a**, **d** PEG-CdTe; **b**, **e** DOX; **c**, **f** PEG-CdTe-DOX. The red fluorescence indicates PEG-CdTe-DOX by arrows (scale bar: 50 μm, × 400; emission wavelengths of DAPI and DOX were 450 and 585 nm, respectively)

**Fig. 4:**
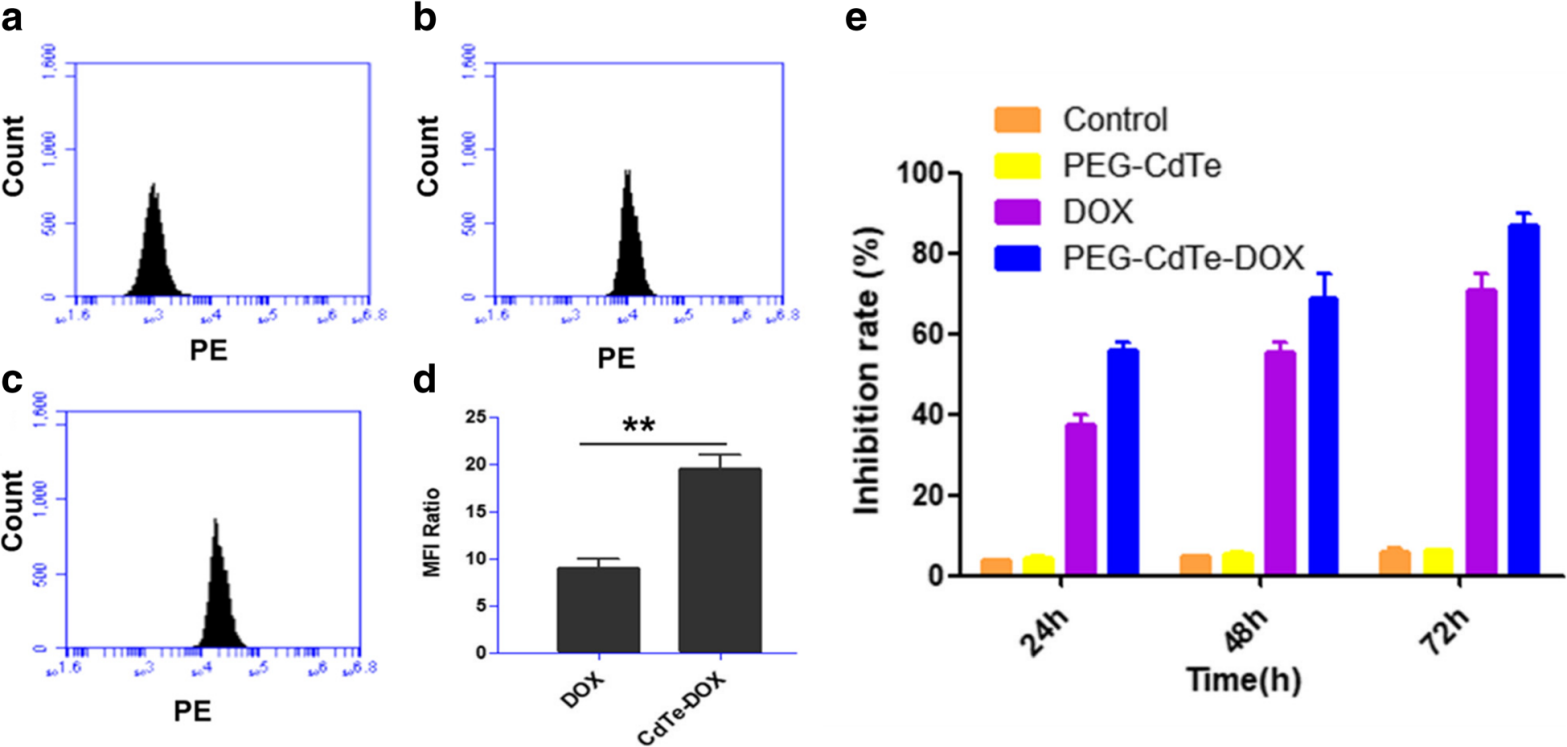
Mean fluorescence intensity in PRMI8226 by flow cytometry and growth inhibition of PRMI8266 cells with different treatments. Notes: **a** PEG-CdTe; **b** DOX; **c** PEG-CdTe-DOX; **d** the fluorescence intensity of DOX and PEG-CdTe-DOX compared to PEG-CdTe (**P < 0.01). **e** CCK-8 analysis of PRMI 8226 cells treated with PBS (control), PEG-CdTe, DOX, and PEG-CdTe-DOX at 24, 48, and 72 h (*P < 0.05)

To determine if PEG-CdTe-DOX can exclusively facilitate DOX accumulation in PRMI 8226 cells, we measured the DOX accumulation by flow cytometry (FCM) of intracellular fluorescence intensity. Intracellular DOX concentration in PRMI8226 cells significantly increased in the PEG-CdTe-DOX group compared with the DOX group (*P* < 0.01, Fig. 4). The cytotoxicity of PEG-CdTe-DOX against PRMI 8226 cells is positively related to the incubation time, which is also longer than in the DOX group (72 h, Fig. 4). The cell viability after incubation for 24, 48, and 72 h was examined using cell counting kit-8 (CCK-8), and the corresponding inhibition rates of PEG-CdTe-DOX were 58%, 70%, and 85%, respectively. The results suggested the viability of PRMI8226 cells in the PEG-CdTe-DOX group significantly decreased compared with the DOX group (*P* < 0.05). Moreover, no significant difference in cell viability was observed between the control group and the PEG-CdTe group. There is no significant difference in cell viability of 397 t cells with PBS and PEG-CdTe (the concentration of PEG-CdTe is the same as that in the RPMI 8226 experiment). These findings are consistent with the intracellular DOX concentrations (Figs. 3 and 4) and indicate PEG-CdTe helps the targeted delivery without interfering with therapeutic effects. Thus, the apoptosis of PRMI 8226 cells enhanced remarkably, indicating PEG-CdTe-DOX could be used as a drug delivery system.

### Apoptosis of PRMI8226 cells

**Fig. 5:**
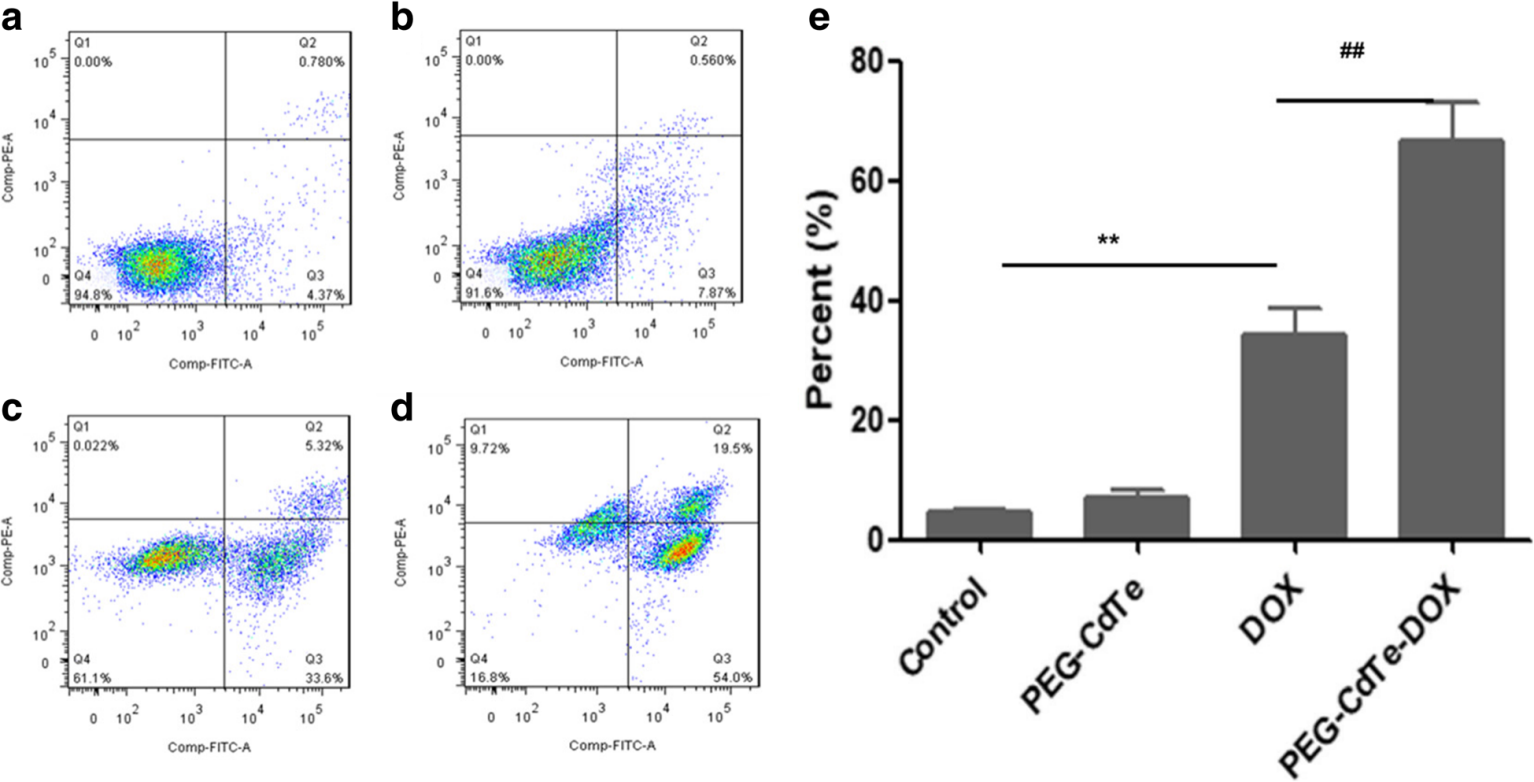
Apoptosis rates of PRMI 8226 cells with different treatments. Notes: **a** PBS; **b** PEG-CdTe; **c** DOX; **d** PEG-CdTe-DOX. **e** Comparative apoptosis rates of PRMI8226 cells in different groups were showed in the bar graph (**P < 0.01 when compared with control; ^##^*P* < 0.01 when compared with DOX)

The total apoptosis rates of PRMI8226 cells measured by FCM were 4.78%, 6.95%, 34.07%, and 66.5% in the control, PEG-CdTe, DOX, and PEG-CdTe-DOX groups, respectively (Fig. 5). The apoptosis rate in PEG-CdTe-DOX group significantly increased compared with the DOX group (*P* < 0.01). However, the apoptosis rates were not significantly different between the control group and the PEG-CdTe group, suggesting that PEG-CdTe was safe and could remarkably increase efficacy of DOX.

### Western Blotting

**Fig. 6:**
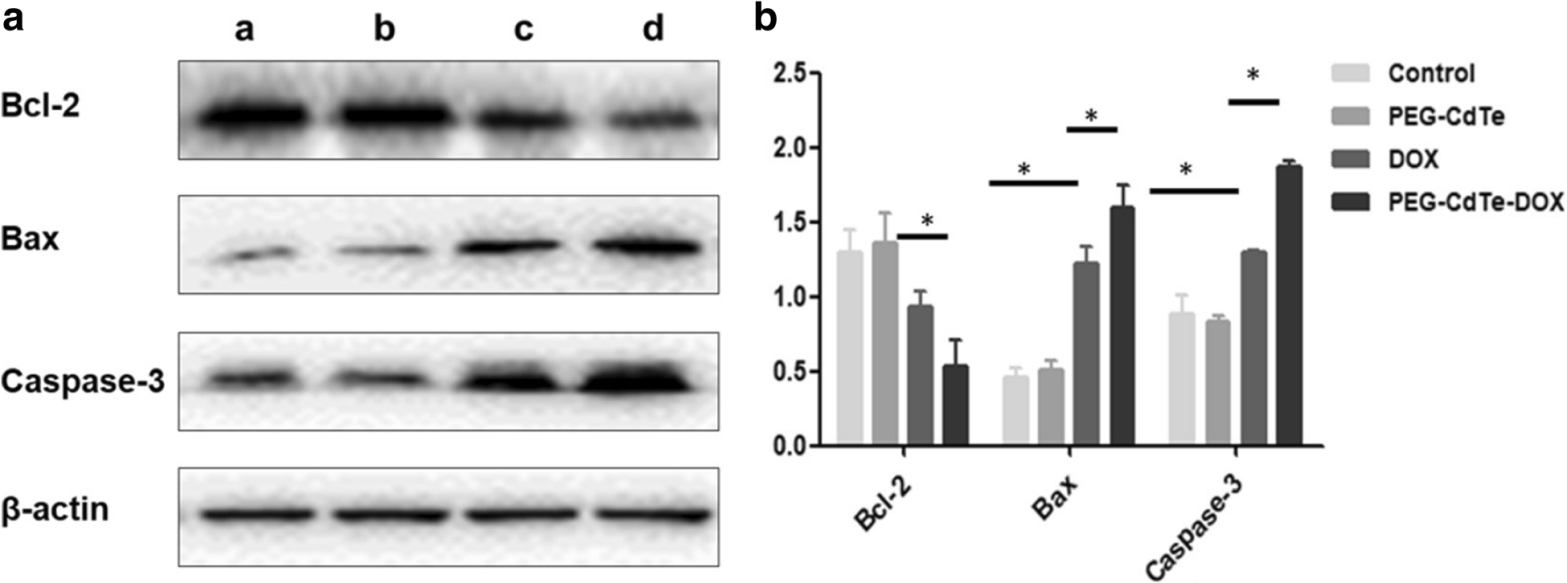
Protein expression of apoptosis-associated genes of PRMI 8226 cells with different treatments. Notes: **a** PBS; **b** PEG-CdTe; **c** DOX; **d** PEG-CdTe-DOX (*P < 0.05)

Western blotting was performed to measure the expression levels of apoptosis-associated proteins Bax, Caspase-3, and Bcl-1 (Fig. 6). Bax and Caspase-3 were gradually upregulated in both the DOX and PEG-CdTe-DOX groups. On the contrast, Bcl2 expression changed reverse. In the PEG-CdTe-DOX group, Bax and Caspase-3 were strongly expressed compared to the DOX group (*P* < 0.05), while the Bcl2 expression was the lowest. These findings confirm that the antitumor activity of PEG-CdTe-DOX is the most effective among the others.

## Discussion

The main cause of tumor chemotherapy failure is the intolerance of patients. The PAD regimen comprising Bortezomib, DOX, and dexamethasone is the first line therapy for multiple myeloma [9]. DOX plays an important role in the treatment of multiple myeloma. DOX inhibits proliferation and induces apoptosis of tumor cells by disrupting DNA [20]. However, cytotoxic drugs such as DOX induce various adverse effects on normal tissues and organs, especially cardiotoxicity [21]. Myeloma is most frequently diagnosed among people aged 65 to 74 years [22], and older multiple myeloma patients often suffer from organ dysfunction and are unable to receive high-dose chemotherapy to improve outcomes. Tolerance is a guarantee for continued treatment of multiple myeloma and thereby restricts the clinical use of DOX. Therefore, numerous EMM patients have no chance of receiving effective treatment because of the failure to stand high-dose chemotherapy. Thus, it is urgent to develop methods that can reduce adverse effects and enhance therapeutic effects. In this work, we developed a DOX-loaded delivery system using PEG-CdTe nanoparticles, which were capable of concentrating drugs selectively in tumor tissues through systemic circulation.

Our study revealed that PEG-CdTe nanoparticles could load DOX with high DL and EE (Fig. 2e), which are consistent with other studies [12, 13]. Moreover, the PEG-CdTe nanoparticles in diameter of 8.2 nm (< 10 nm) could be quickly metabolized by the urinary system [23], and PEG-CdTe-DOX nanoparticles in size of 78.31 nm (< 200 nm) prolonged the blood circulation time and reduced or even avoided plasma opsonization and absorption in the reticuloendothelial system [23]. As a nanoparticle carrier, the drugs were released from PEG-CdTe in a pH-triggered and pH-controlled pattern and prolonged the circulating period, which were important for a practical drug delivery system to prevent unwanted immune response and tissue reactions against the drug carrier [24]. The tumor microenvironment was under lower pH compared with normal tissues because of the hypoxic state [25], and thus released more DOX. The drug concentration in acidic microenvironment (tumor tissues), and thereby the efficacy of chemotherapeutic drugs, can be improved [26]. Hence, the drug was massively released around the tumor cells, whereas the drug was mostly remained in the carrier in the normal physiological environment and was less released to normal tissues. Intracellular experiments also confirmed that more DOX was delivered to PRMI 8226 cells. Therefore, the chemotherapeutic efficacy was improved and the adverse effects on normal tissues were induced.

Experiments in vitro consistently showed that PEG-CdTe-DOX targeted tumor cells and increased drug accumulation there (Fig. 3). Similarly, the proliferation inhibition and apoptosis of PRMI 8226 cells were increased by PEG-CdTe-DOX compared to DOX alone at the same concentration (Fig. 4). Therefore, lower DOX was required to achieve the same chemotherapeutic efficiency. In addition, the RPMI 8226 cell proliferation inhibition and apoptosis induction can be increased by PEG-CdTe-DOX, which is the main way the drugs kill myeloma cells (Fig. 5). Furthermore, no significant difference was found between the PEG-CdTe group and the control group, which confirmed PEG-CdTe had no therapeutic activity in vitro. What should be noted is that our results indicate the high safety and low cytotoxicity of PEG-CdTe at low concentration, which is consistent with other studies [12, 27]. However, the apoptosis rate of PMIR 8226 cells evaluated by FCM in the PEG-CdTe-DOX group is obviously higher than the DOX group (*P* < 0.01). Thus, PEG-CdTe could significantly attenuate the side effects of DOX by delivering the drugs to tumor cells.

The three main pathways of apoptosis were considered: the mitochondrial pathway, the endoplasmic reticulum pathway, and the death receptor pathway. In the mitochondrial pathway, apoptogenic factors (e.g., cytochrome C) are first released from the mitochondria into the cytosol, and then initiate the apoptosis of this pathway induced by the caspase pathway [28]. The Bcl2 family consists of pro-apoptotic protein (Bax, Bad, and Bak) and anti-apotoptic proteins (Bcl-xl and Bcl2) [29]. Bax can migrate from the cytosol to the mitochondrial membrane when stimulated by apoptosis signals, which is the apex of the “life or death” cellular mechanisms. Bcl2 and Bax are a pair of positive and negative gene regulations, as Bax induces apoptosis while Bcl2 inhibits it [30]. Caspase-3 may not only promote apoptosis, while surviving is the strongest inhibitor of apoptosis, but also promotes cell proliferation, which can directly in activate caspase-3 and caspase-7, thereby blocking the apoptosis [31, 32]. Caspase-3, an executioner in the caspase family, can also cleave and inactivate poly-adp-ribose polymerase when initiated [12]. In the present study, the expressions of related apoptotic proteins were detected to explore the molecular mechanism how PEG-CdTe-DOX enhanced antitumor activity. It was found that PEG-CdTe-DOX can increase the cytotoxic effects on tumor cells by inducing apoptosis via the caspase-mediated apoptotic pathway.

## Conclusions

We fabricated the PEG-CdTe-DOX for EMM treatment with high EE and DL. This system can deliver DOX to RPMI 8226 cells in a targeted way, thereby increasing the therapeutic effects and declining the side effects of DOX. The targeting of PEG-modified CdTe could increase the chemotherapeutic effectiveness and reduce the side effect, which may pave the way for better cancer treatment.

## Abbreviations

DAPI: 4′,6-diamidino-2-phenylindole
DL: Drug loading
DOX: Doxorubicin
EE: Encapsulation efficiency
EMM: Extramedullary multiple myeloma
FCM: Flow cytometry
HPLC: High-performance liquid chromatography
MM: Multiple myeloma
PBS: Phosphate buffer saline
PEG-CdTe-DOX: Polyethylene glycol-modified cadmium telluride quantum dots
SDS/PAGE: Sodium dodecyl sulfate polyacrylamide gel electrophoresis
TEM: Transmission Electron Microscope
